# Original Personalized Reconstruction Method for Certain Large Tumors of the Lower Lip (Not Including Oral Commissures)

**DOI:** 10.3390/medicina61010004

**Published:** 2024-12-24

**Authors:** Ovidiu Dan Grigorescu, Marius Irimie, Nadinne Roman, Roxana Steliana Miclaus, Maria-Cristina Cîmpeanu, Bogdan-Radu Necula, Christian-Gabriel Strempel, Simona Grigorescu, Marius Alexandru Moga, Dana Gabriela Festila

**Affiliations:** 1 Faculty of Medicine, Transilvania University of Brașov, 29, B-dul Eroilor, 5000036 Brașov, Romania; ovidiu.grigorescu@unitbv.ro (O.D.G.); marius.irimie@unitbv.ro (M.I.); nadinneroman@unitbv.ro (N.R.); roxileta2009@yahoo.com (R.S.M.); cristinamateescu91@yahoo.com (M.-C.C.); bogdan.necula@unitbv.ro (B.-R.N.); moga.og@gmail.com (M.A.M.); 2 Faculty of Economic Sciences and Business Administration, Transilvania University of Brașov, 29, B-dul Eroilor, 5000036 Brașov, Romania; christian.strempel@student.unitbv.ro; 3 Faculty of Dental Medicine, University of Medicine and Pharmacy “Iuliu Hatieganu”, 8, Victor Babes Str., 400347 Cluj-Napoca, Romania

**Keywords:** lower lip defects, tumors, personalized reconstruction, original method, associated local flaps

## Abstract

*Background/Objective*: Surgical treatment of extensive tumors of the lower lip generates important defects in its structure and functions. Over time, a multitude of reconstruction options for these defects have been imagined. Unfortunately, the majority involve the use of both local and regional flaps, which either lack labial structure or disorganize the oral commissures as nodal oral function points. We set out to design a new reconstruction method that is not burdened by any of the above disadvantages, starting from the necessity to reconstruct large lower lip defects by exclusively using local and/or regional labial flaps belonging only to the healthy upper lip and the remaining structures of the lower lip after surgical tumor removal. In this way, the tissues involved in lower lip reconstruction will have a 100% labial structure and, supplementarily, the remaining oral commissures will not be damaged. *Method*: This new reconstruction method is based on the original association of WY-plasty to reduce the size of the large primary defect until it becomes congruent with the Abbe–Sabattini cross-lip flap. *Results*: Applied in a personalized manner, impressive results were obtained in several patients with lip tumors affecting about one-half of the lower lip length and in whom oncological surgical ablation produced defects in more than two-thirds of it. *Conclusions*: This new method is characterized by functional oral advantages, and can be applied in a personalized way to only certain categories of patients. Other disadvantages are characteristic and specific of Abbe–Sabattini cross-lip flap plasty.

## 1. Introduction

The lips have crucial importance not only for esthetic appearance but also for functional roles in speech, mastication, and facial expression. This is why the reconstruction of lip defects represents a complex challenge in the fields of plastic and reconstructive surgery. These defects can be caused by a variety of factors, including trauma or surgical interventions for tumor excision [1]. The primary goal of reconstruction is to restore oral competence, maintain the patency of the oral aperture, and restore anatomical relationships, both static and dynamic (as in smiling, for example) [2]. Therefore, reconstruction techniques must be carefully chosen to ensure a balance between functionality and esthetics.

Over the years, numerous reconstruction methods have been developed, ranging from simple primary suture techniques to complex free tissue transfers. Each method has specific advantages and disadvantages, and the choice of the optimal technique depends on factors such as the size and location of a defect, a patient’s age, and their overall health condition [3].

Malignant tumors of the lips, and especially those of the lower lip, have become an increasingly common pathology that has led plastic and reconstructive surgery specialists to find new methods to provide increasingly effective and personalized surgical treatments.

The effectiveness of surgical treatment in oncological pathology is currently based on two important principles that must be applied to most surgically treated cases: (a) the absolute necessity of “oncological” ablation in absolutely clinically healthy tissue and (b) the obligation to ensure a high degree of both structural and functional reconstruction of the defects created by an oncological excision.

In the case of extensive malignant tumors of the lower lip involving half (or more) of its length, but not including oral commissures, the greatest difficulty is represented by the reconstruction of the defect created by a wide oncological ablation, avoiding (as the main condition) the impairment of its commissural structure and functions. After the removal of the tumor, this defect usually represents about two-thirds (sometimes even more) of the length of the lower lip, involving not only its structural reconstruction but, much more importantly, its specific functions.

Plastic surgeons have devised, over time, many methods intended to solve these large defects (not including oral commissures) in order to allow both structural and functional reconstruction, as will be emphasized in the Section 4—Discussion.

**Scheme 1:**
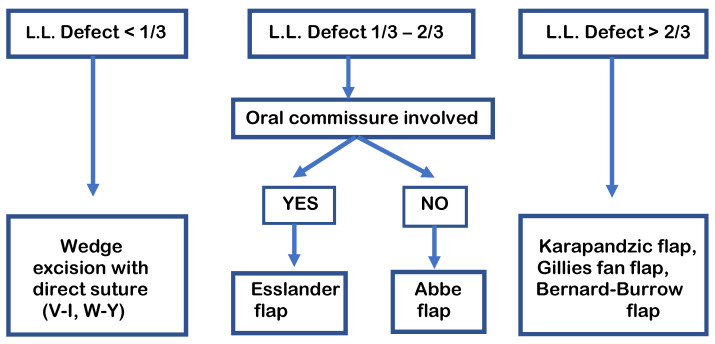
Classic algorithm for choosing the optimal method in the reconstruction of lower lip (L.L.) defects.

The majority of these methods used local and regional flaps, which either disorganize the oral commissures as nodal points of labial function or affect the donor areas functionally and esthetically. For these reasons, the vast majority of methods imagined and used up to now have important limits and cannot be applied without incurring risks assumed by both the surgeon and the patient (Scheme 1).

For these reasons, the development of a method that is not burdened by any of the above disadvantages can obviously become a research priority for us. At the same time, we were energized in this research by the fact that the emergence of a new method will allow for a personalized and effective approach to specific types of lip and oral defects.

## 2. Materials and Methods

### 2.1. Hypothesis and Purpose of the Work

To succeed in identifying a new reconstruction method in cases of large lower lip defects arising as a consequence of the ablation of malignant tumors, we formulated, as a starting hypothesis, the compulsory condition to use only local and/or regional flaps characterized by having an exclusively labial structure. It is clear that these types of flaps only belong to the intact upper lip and to the remaining structures of the lower lip after tumor surgical ablation. In this way, the main advantage of the new procedure is that the tissue material utilized for the reconstruction will have a 100% labial structure. A secondary, but mandatory, requirement regarding the absolute preservation of both oral commissures during lower lip reconstruction was also defined, characterized by the explicit interdiction to not damage either of the two oral commissures in any way.

**Figure 1:**
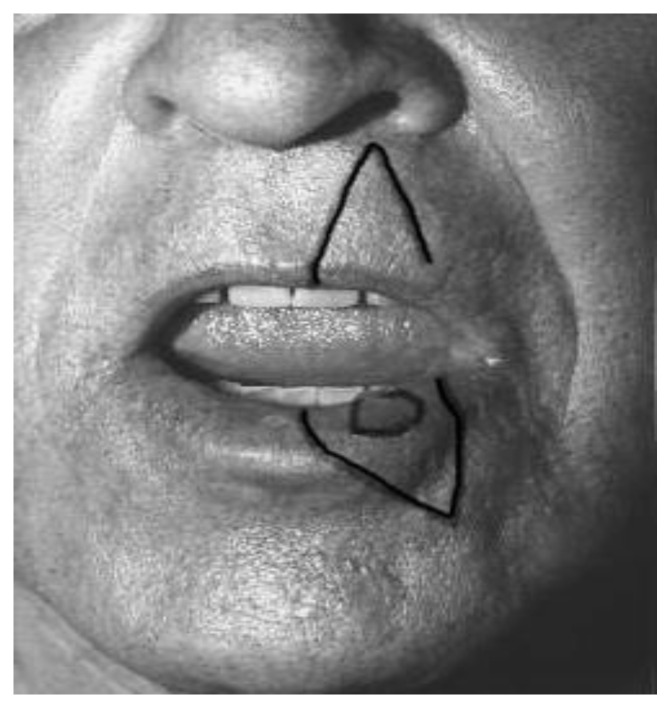
The Abbe–Sabattini flap could be used for defects of the lower lip no larger than one-third to one-half of the lip length (personal theoretical design).

**Figure 2:**
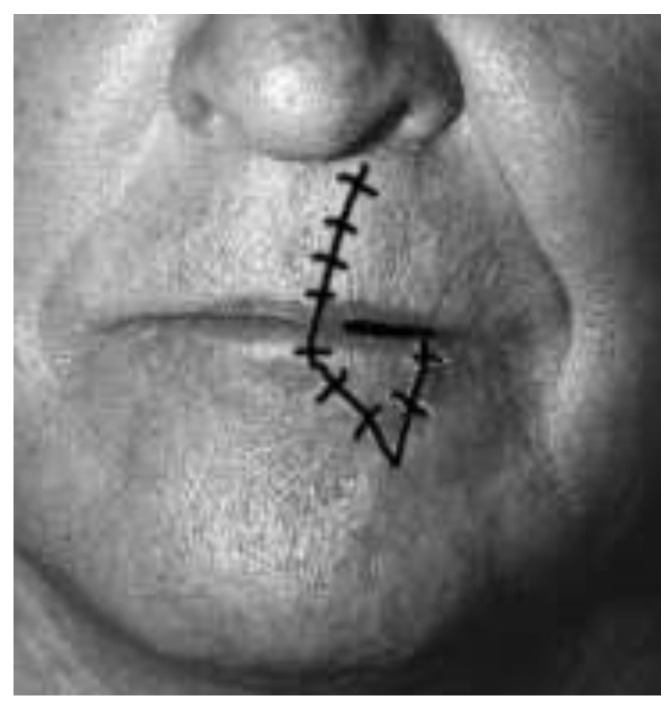
The Abbe–Sabattini flap is transferred from the upper lip to the lower lip defect without the involvement of any oral commissures (personal theoretical design).

A theoretical model of the new method was built based on the following logical sequence:

(a) The perfect flap in terms of structure and function for the reconstruction of lower lip defects no larger than half of the length of the lip and not involving the integrity of the oral commissures seems to be the Abbe–Sabattini cross-lip flap.
  As can be easily seen in Figure 1, the oncological ablation of a small/medium malignant tumor in terms of length in the lower lip can generate a defect of about one-third of the structure of the lower lip.
(b) Plasty with the Abbe–Sabattini cross-lip flap can be performed to reconstruct only small/medium labial defects, sized from about one-third to one-half of the lower lip length. This type of defect can be reconstructed by using a triangular flap created at the upper lip level and rotated towards the lower lip, with immediate closure of the newly created upper lip defect by a direct suture, as indicated in Figure 2.

Observation: Along with the images in Figure 1 and Figure 2 (representing a personal drawn theoretical design of the presented method), the effective way of performing this type of plasty will be presented as clinical case in the following (Figure 3, Figure 4, Figure 5, Figure 6, Figure 7, Figure 8 and Figure 9).

(c) Similar small/medium defects created in the lower lip via the removal of benign/malignant tumors can be canceled by performing WY-plasty.

**Figure 10:**
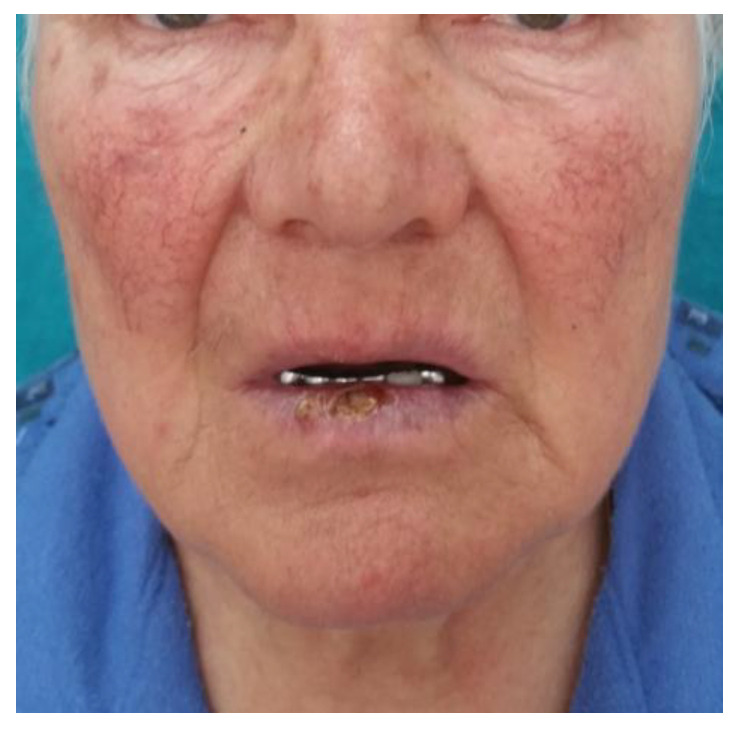
Lower lip malignant tumor (one-third–two-fifths of lower lip length) (personal case image).

**Figure 11:**
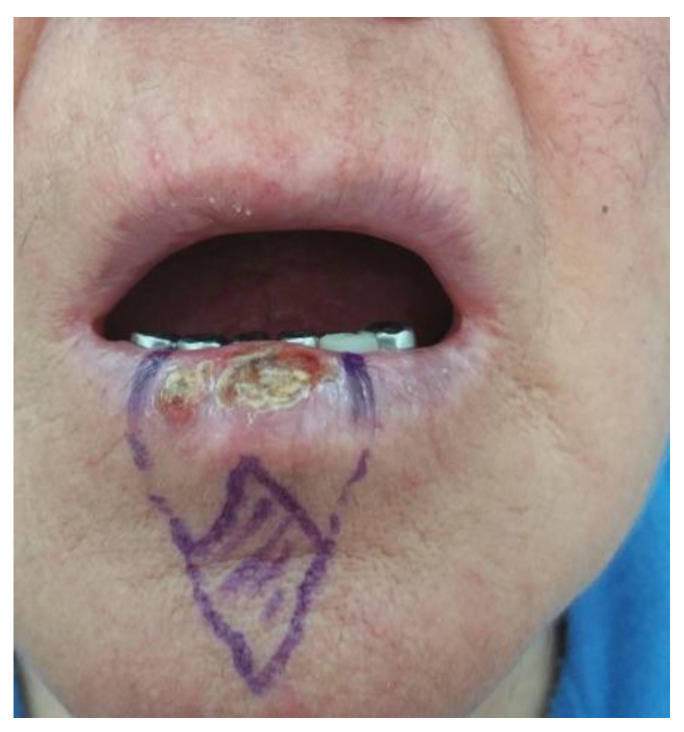
The design of the WY-plasty on the lower lip (personal case image).

**Figure 12:**
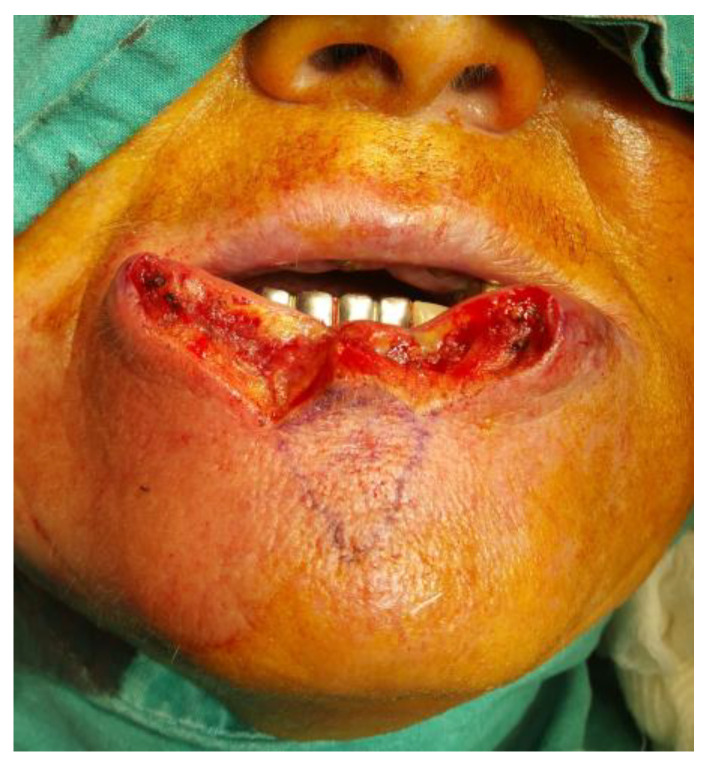
Lower lip malignant tumor with 40% (two-fifths) of the lip length removed (personal case image).

**Figure 13:**
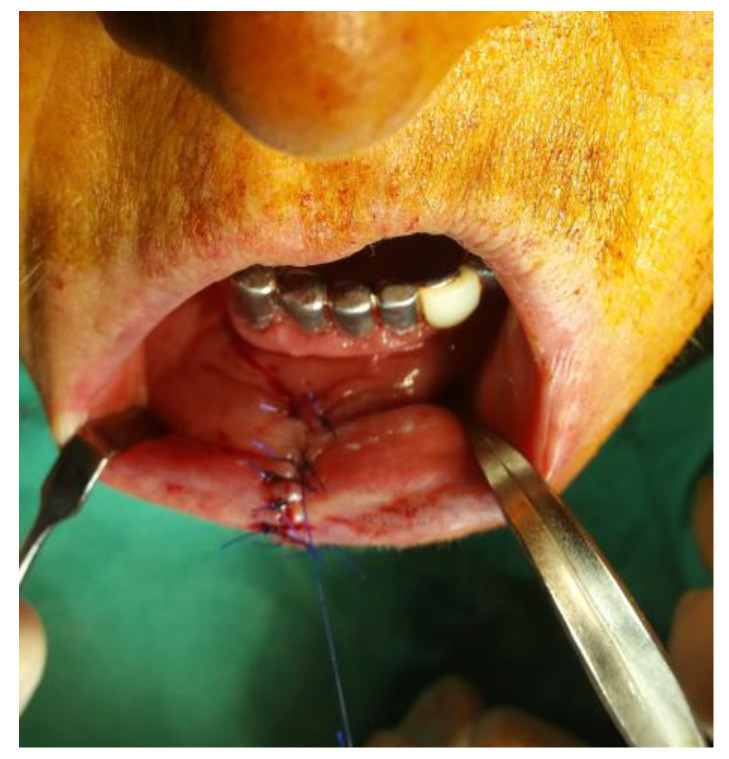
The intra-oral result of performing WY-plasty on the lower lip (personal case image).

**Figure 14:**
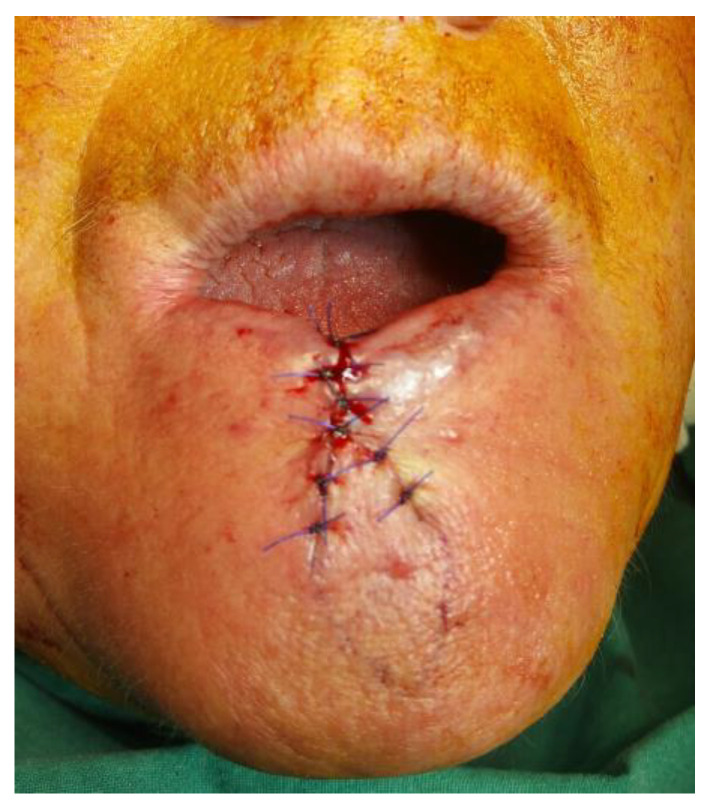
The immediate external result of performing the WY-plasty on the lower lip (personal case image).

**Figure 15:**
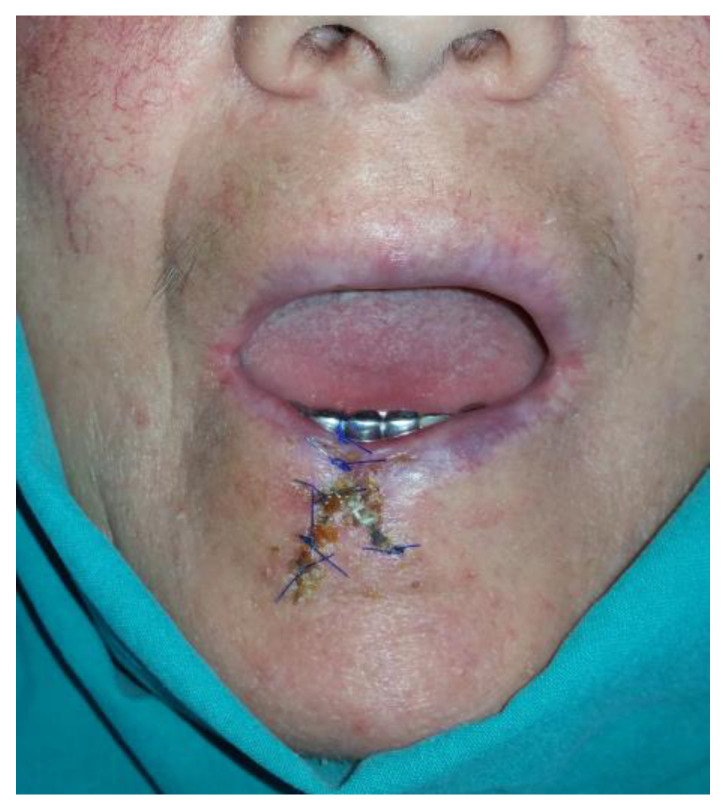
The late result (14 days post-op) of performing the WY-plasty on the lower lip (personal case image).

In all situations where defects of the lower lip narrower than one-half of its length are generated, tumor resection in a “W” shape allows both oncological ablation and relatively easy reconstruction of the resulting defect, due to the superior elasticity of the lower lip structures and an important tissue reserve of it, as shown in Figure 10, Figure 11, Figure 12, Figure 13, Figure 14 and Figure 15.

These two different methods (Abbe–Sabattini cross-lip plasty and WY-plasty) seem to be similar in terms of the following aspects:

- The width of the resulting lower lip defect after tumor resection;
- The quality of the reconstructed lower lip, having the advantage of repairing defects with similar tissue;
- The preservation of the structure and functions of both oral commissures, whose integrity is not affected by performing either of these two methods.

In this context, we asked ourselves whether the association of these two methods would not allow for the removal of much more important defects in the length of the lower lip in the case of extensive or neglected malignant tumors.

Following a logical sequence of principles and measurements, we finally found that the association of the two methods analyzed above can allow for the reconstruction of extensive defects of the lower lip, starting from two-thirds of its length and sometimes, in particular conditions, more than that.

### 2.2. Analysis of the New Method

Starting from the already presented hypothesis, we devised an original method that allows for the removal of large defects in the lower lip and the functional reconstruction of this complex structure. This new method consists of the association of the two types of labial plasties (WY-plasty with the Abbe–Sabattini cross-lip flap plasty) in order to simultaneously use the maximal reconstructive potential of each of these methods. Thus, the remaining defect after the oncological resection of an extensive tumor (two-thirds of the lower lip length or more) will be partially covered and reconstructed in the first stage by the effect of WY-plasty. The remaining defect, much smaller than the primary one, will be covered and reconstructed through the second procedure (the Abbe–Sabattini cross-lip flap) performed in the same stage after WY-plasty, as the last part of the first surgical intervention.

**Figure 16:**
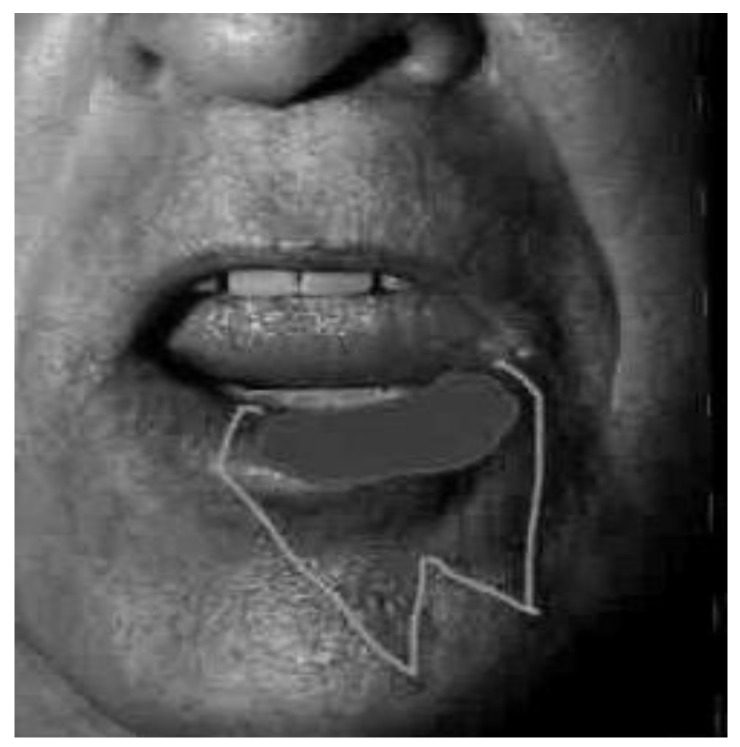
Extensive tumor on two-thirds of the lower lip length with wide resection in a “W” shape (personal theoretical design).

**Figure 17:**
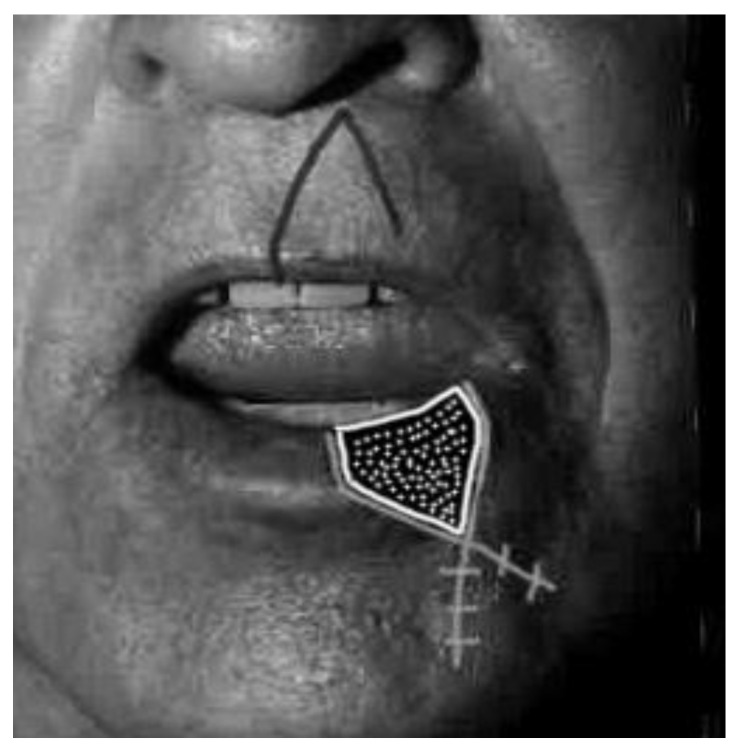
Association of WY-plasty with the Abbe–Sabattini cross-lip flap in wide defects of the lower lip (personal theoretical design).

The theoretical model of the new method was built and presented below (Figure 16 and Figure 17).

The explicit demonstration of the proposed innovative method will be achieved by presenting the case of one of the patients included in the study.

Case history

The patient (M.A., 76-year-old female, smoker, no other known diseases, village resident, living alone, no relatives, no family members) declared that the tumor process evolved slowly, for 6 years. During this time, she did not consult any doctor (the village lacking a GP family doctor). This situation allowed the patient to neglect the evolution of the lower lip tumor, which developed explosively in the last 6 months before her admittance into the hospital and associating pain and small hemorrhages that stopped spontaneously as specific symptoms. Additional investigations (CT scan, US scan) did not reveal local or regional metastases, but only local inflammatory tissular micro reactions.

Informed consent could not be obtained to use the classical methods already known in the literature, the patient explicitly insisting that she does not agree with the disorganization of the oral commissures, especially because she was wearing removable dentures. The surgical intervention was performed under general anesthesia and lasted 165 min, of which the first 75 were used for tumor ablation and surgical inspection of the anterior cervical, chin and submandibular regions.

**Figure 18:**
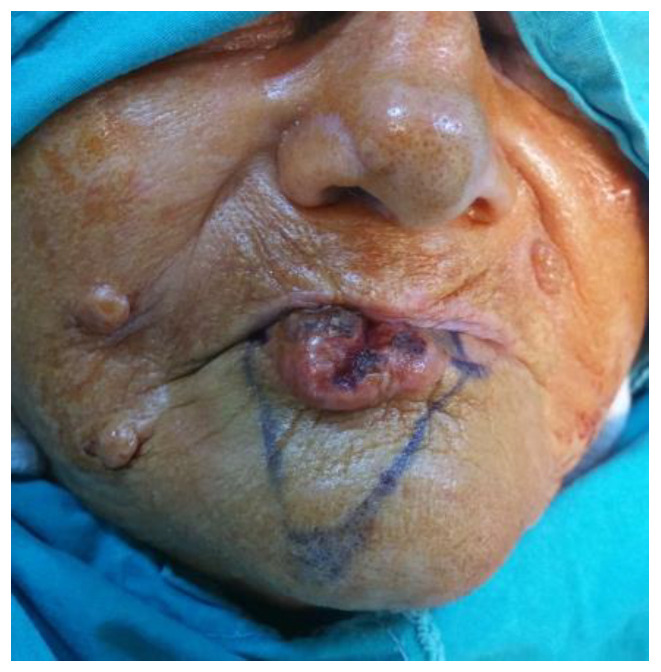
The marks for classical “V” excision (60° angle).

**Figure 19:**
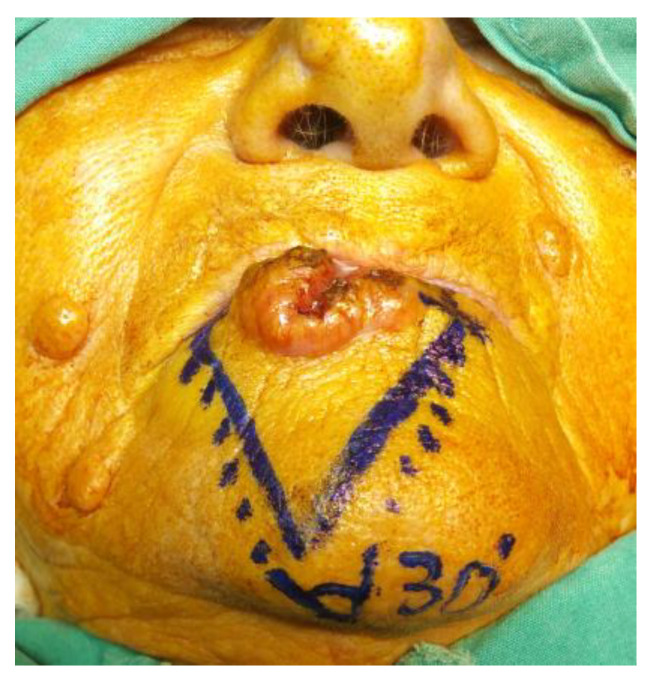
The marks for modified “W” excision (30° angle).

**Figure 20:**
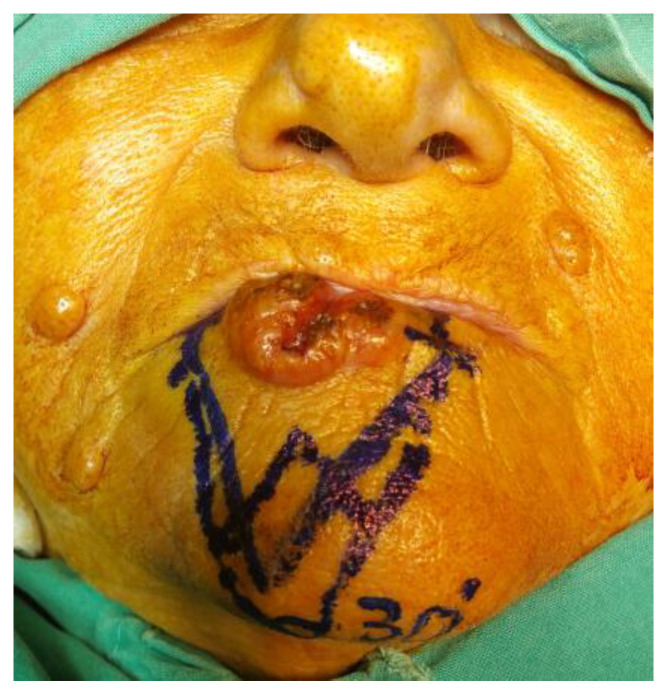
The marks for “W-V” excision (30° angle).

**Figure 21:**
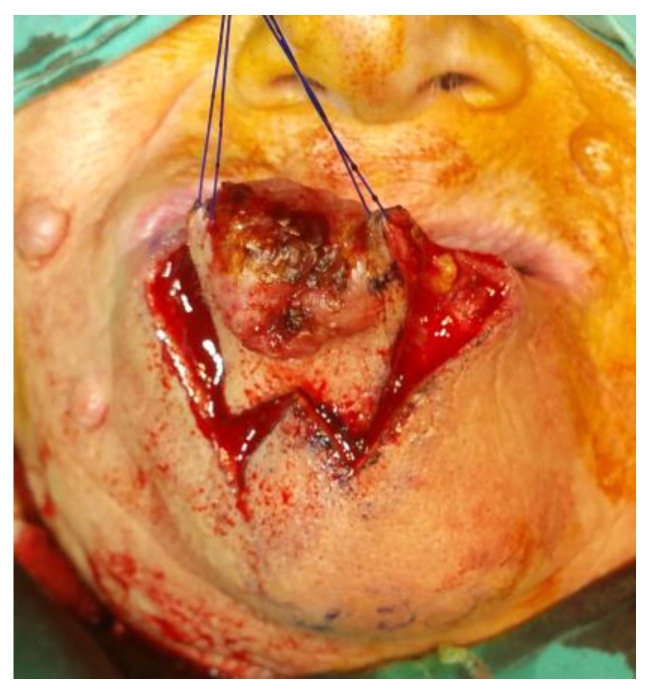
The tumor “W” skin excision with tissue preservation of the angle top region.

**Figure 22:**
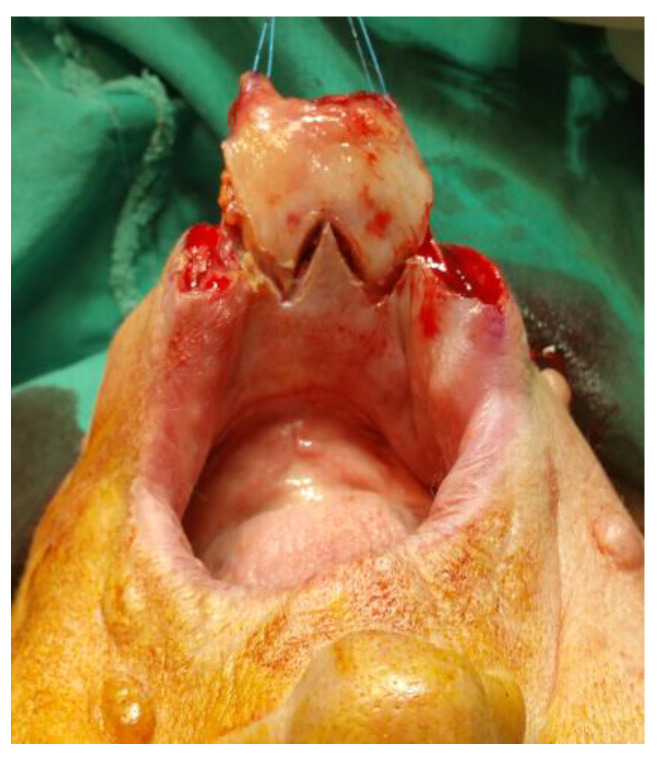
The “W” mucosal incision for tumoral excision.

**Figure 23:**
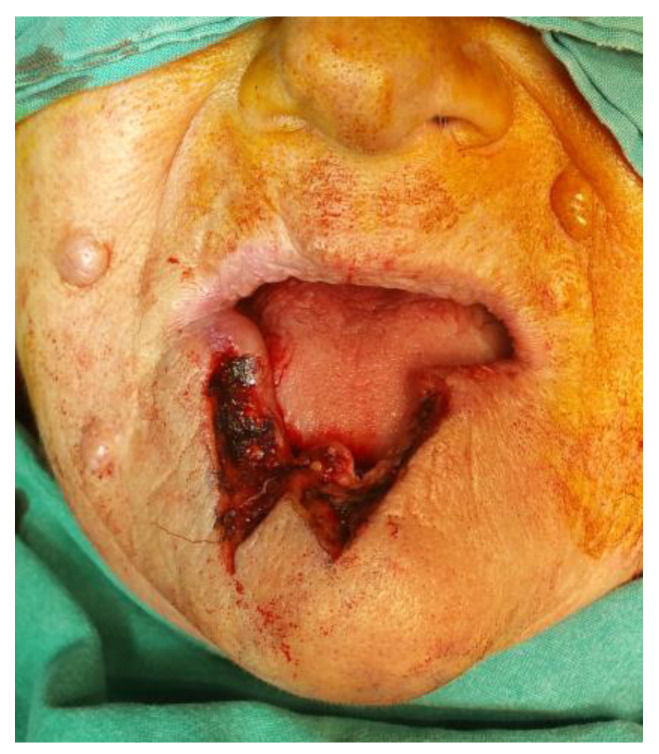
The “W” large defect of the lower lip.

The surgical technique consisted in the ablation of the tumor using initially a wide W-shaped incision at the level of the lower lip and chin region, followed by incisions made perpendicular to the longitudinal axis of the lower lip, approximately 1.5 cm from the macroscopic limit of the tumor tissue, on both sides of it and completed by the W-shaped incision at the vestibular mucosa level (Figure 18, Figure 19, Figure 20, Figure 21, Figure 22 and Figure 23).

**Figure 24:**
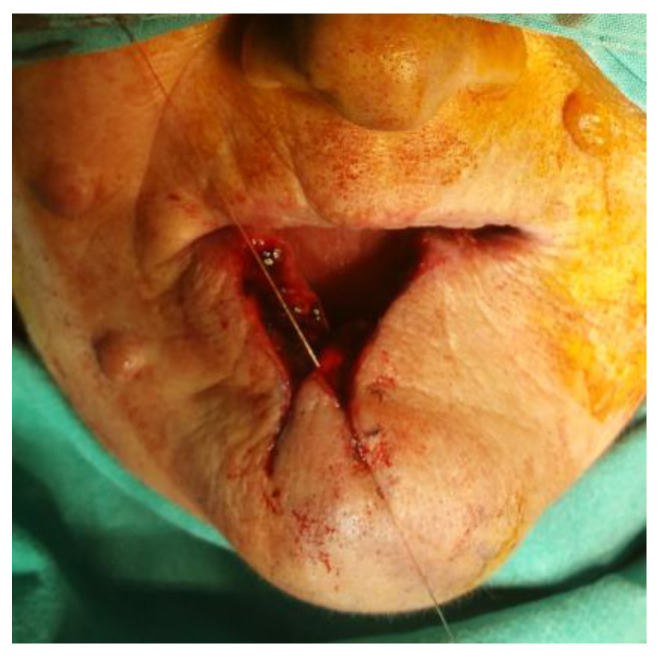
The initial partial closure causing the defect to be halved.

**Figure 25:**
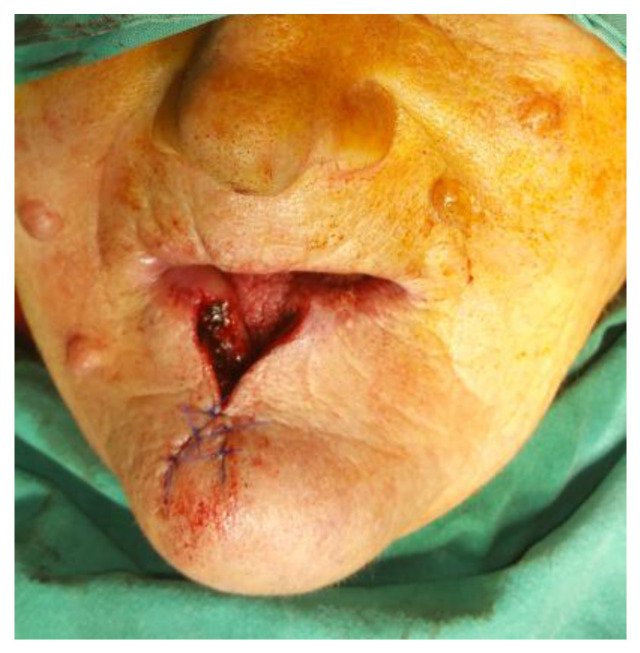
The halved defect (“V”shape) to be solved by Abbe-Sabattini cross-lip plasty.

The defect was partially closed by performing the first stage of the W-Y plasty (Figure 24 and Figure 25).

**Figure 26:**
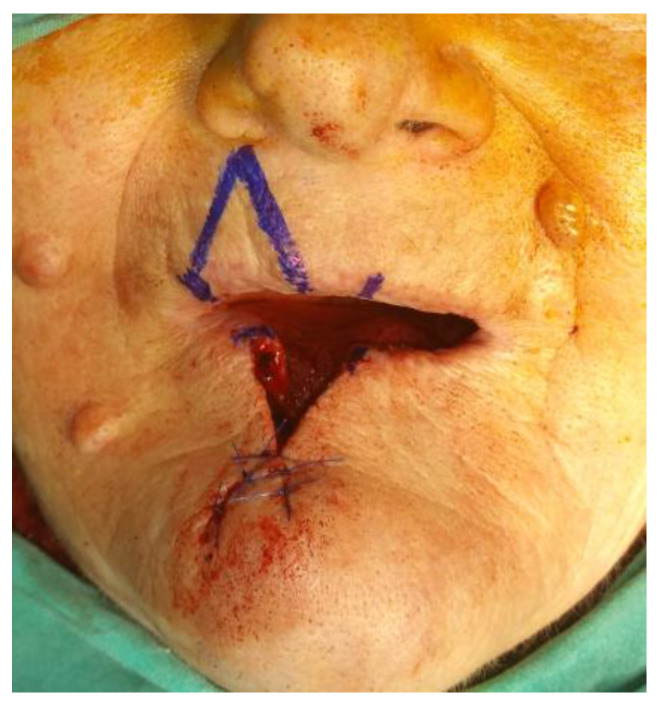
The “V” marks for cross-lip plasty with the vascular pedicle medially tailored.

**Figure 27:**
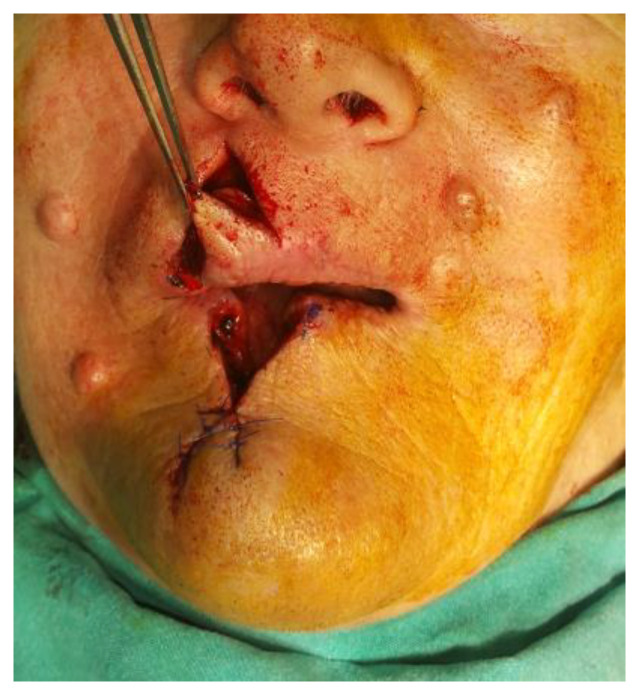
The release of the tailored upper lip flap generating the secondary upper lip defect.

**Figure 28:**
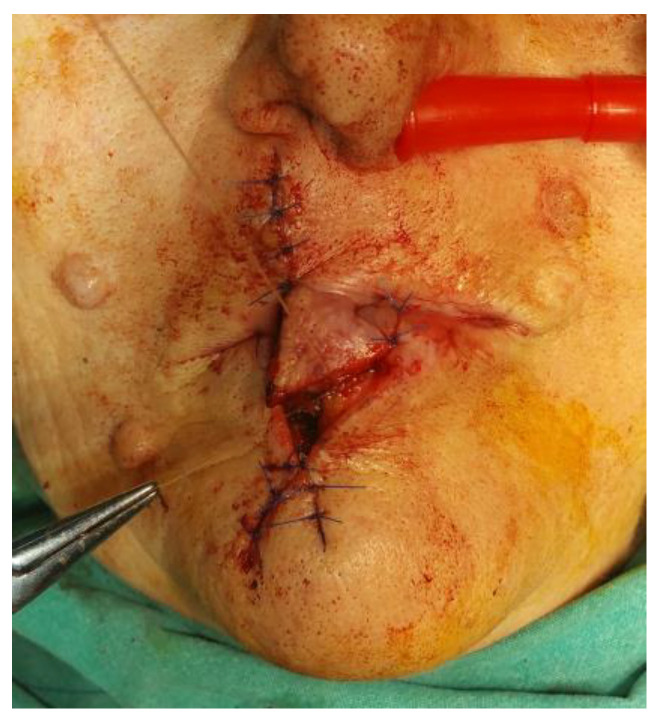
The closure of the upper lip defect (“I” shape) with lower lip length reduction (one-third).

**Figure 29:**
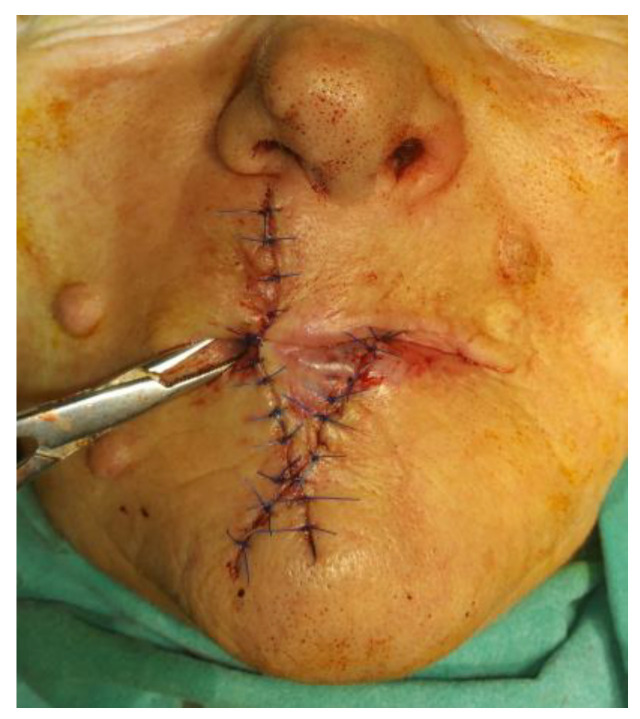
Finalizing the cross-lip plasty by closing the “V” shape lower lip defect with the upper lip “V” shape cross-flap (small right mouth cleft).

The remaining defect at the level of the lower lip was cancelled by using the Abbe-Sabattini plasty, involving a cross-lip flap (Figure 26, Figure 27, Figure 28 and Figure 29).

**Figure 30:**
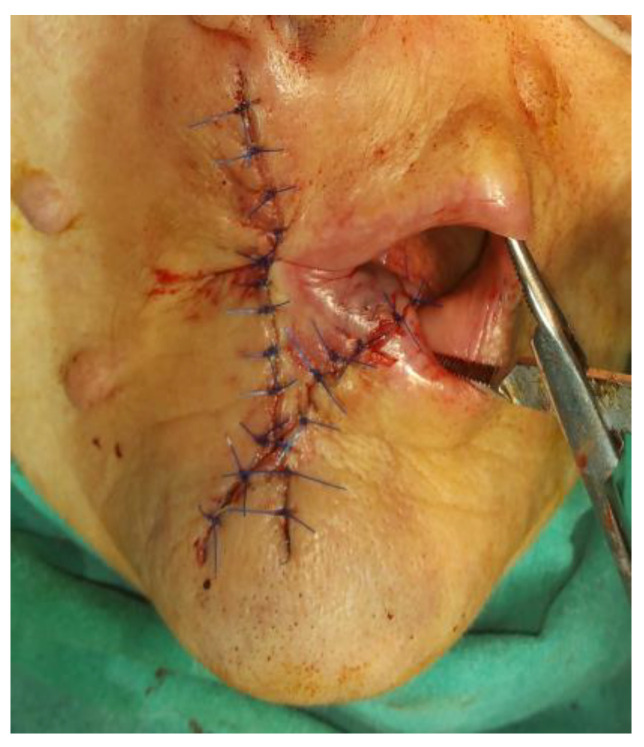
The division of the labial cleft closure into two smaller and unequal clefts (the larger one as indicated in the image as left mouth cleft).

Two unequal mouth clefts were generated (Figure 29 and Figure 30).

**Figure 31:**
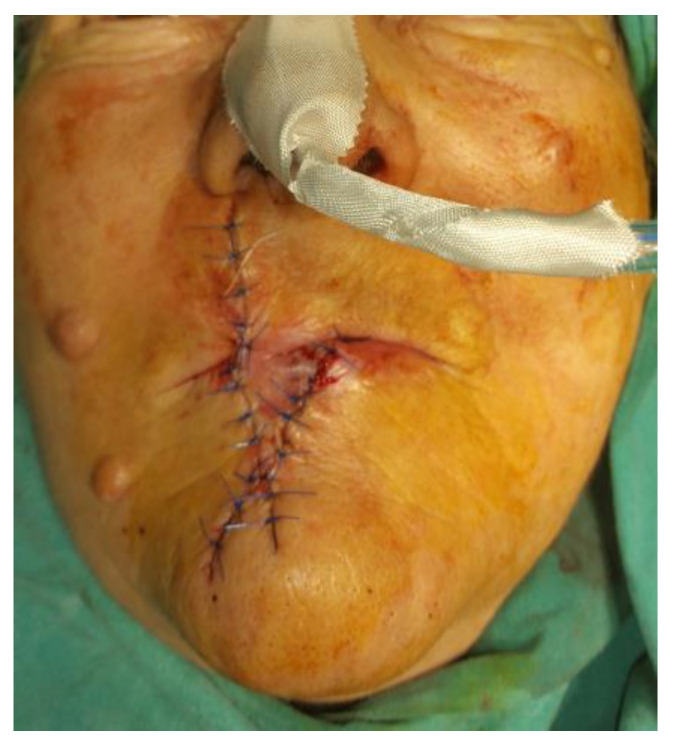
Finalized sutures of the cross-lip plasty by “W-V-V-I” sutures (nasogastric tube in site).

A nasogastric tube was mounted during the intervention, to ensure the patient’s nutrition in the following 2–3 weeks postoperatively (Figure 28 and Figure 31).

The following 3 weeks, a specific nursing was performed for the surgical wounds, then, after 14 days, the suture stiches were removed.

**Figure 32:**
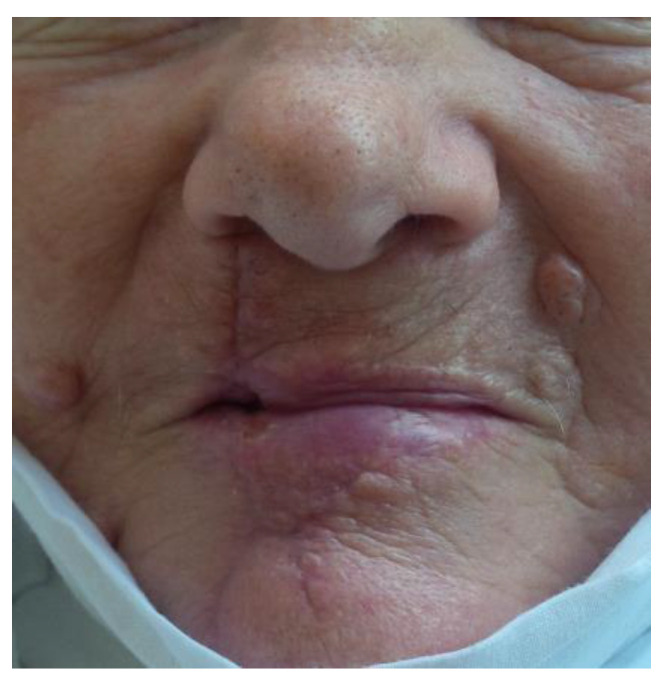
The late result of innovative method (6 months postop).

**Figure 33:**
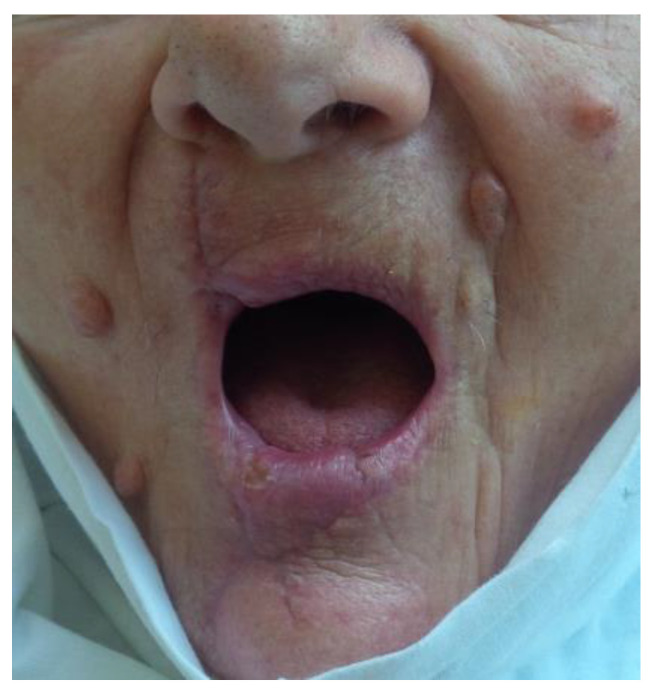
The late result of innovative method (6 months postop).

After 3 weeks from the first stage of the intervention, the second stage of the Abbe-Sabattini plasty was performed by integrating of the upper lip flap into the new structure of the reconstructed lower lip defect. The late result (represented by the 6 months postoperative situation) is presented (Figure 32 and Figure 33).

In the previous illustrations, the sequence of the stages of the surgical treatment of an extensive malignant tumor of the lower lip can be explicitly observed using the original method described above.

In conclusion, the clinical case of the previous presented female subject, 76 years old, with a large tumor of the lower lip (more than one-half of the lip length) demonstrates (Figure 18, Figure 19, Figure 20, Figure 21, Figure 22, Figure 23, Figure 24, Figure 25, Figure 26, Figure 27, Figure 28, Figure 29, Figure 30, Figure 31, Figure 32 and Figure 33) that a tumor extending over about 50% of the lower lip length and requiring a large/oncological resection of about two-thirds of the lower lip could be removed in oncological safety limit conditions and finalized by lower lip reconstruction through the new/innovative method. Starting with a “W”-type excision (Figure 21, Figure 22 and Figure 23), on the lower lip is shown the way in which the defect created by the oncological resection is partially canceled by performing local WY-plasty (Figure 24 and Figure 25). Similarly, the way in which the Abbe–Sabattini cross-lip flap is performed in order to cancel the rest of the defect remaining after finalizing WY-plasty can be observed regarding the upper lip (Figure 26, Figure 27, Figure 28, Figure 29, Figure 30 and Figure 31).

**Figure 34:**
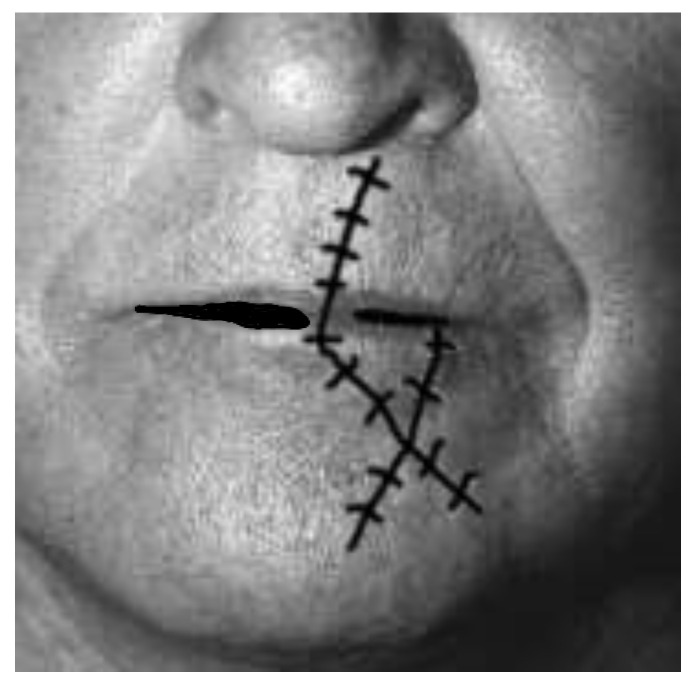
The modeled first postoperative situation after the first stage, with the two unequal oral clefts (personal theoretical design).

**Figure 35:**
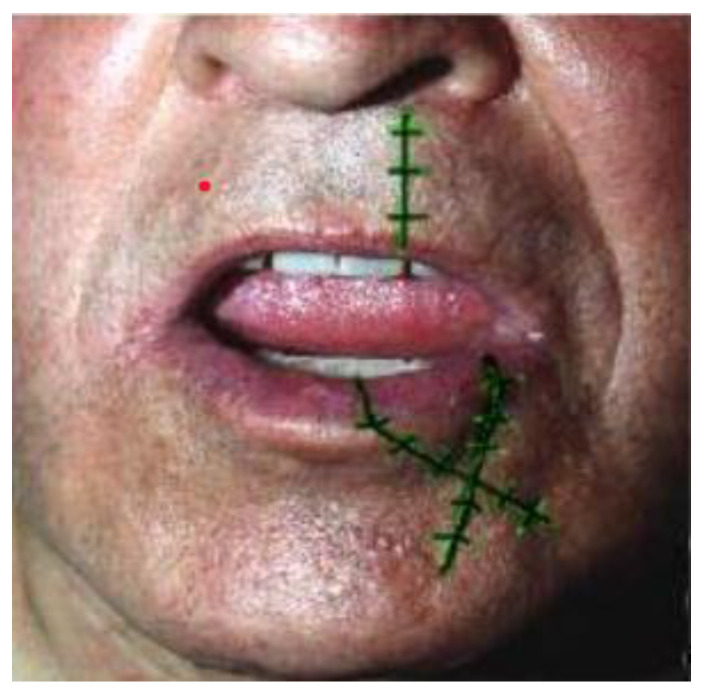
The modeled situation after the last postoperative stage of new-method-associated plasties (personal theoretical design).

**Figure 36:**
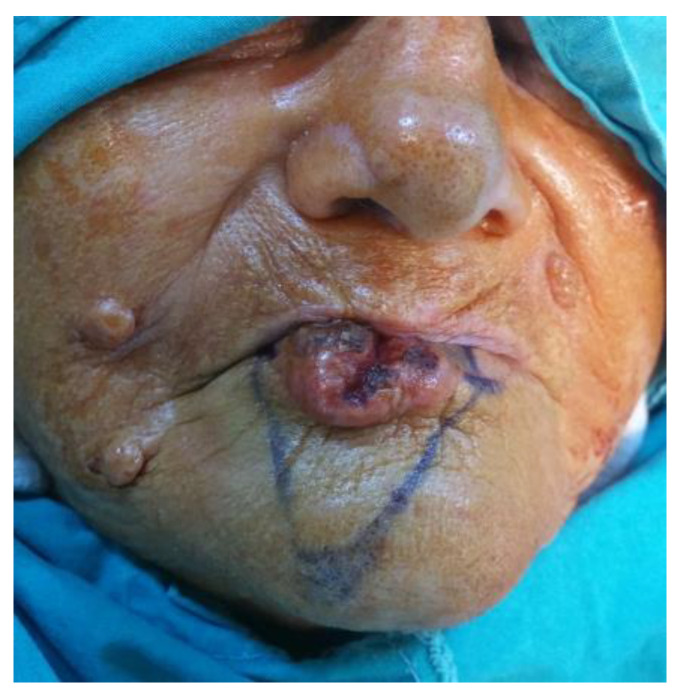
The initial stage of a lower lip large tumor (M.A., 76-year-old female).

**Figure 37:**
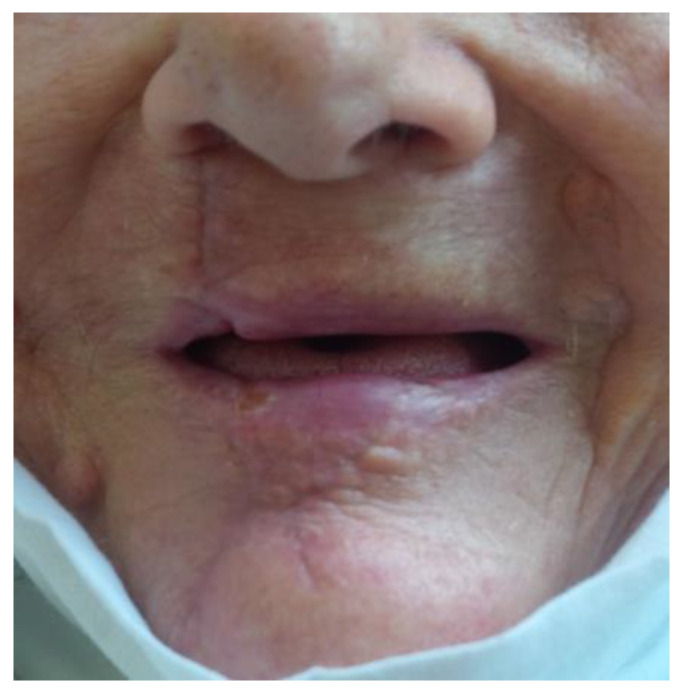
The result of innovative approach for a large tumor of the lower lip (M.A., 76-year-old female).

The association of the two types of reconstruction methods will finally lead to the situation presented as theoretical design in Figure 34 and intraoperative images in Figure 29 and Figure 30, in which the labial cleft is divided into two smaller and unequal clefts due to the effect of the presence of the still-not-sectioned pedicle of the Abbe–Sabattini cross-lip flap. As shown, both oral commissures remain structurally untouched. A modeled situation of the reconstructed lip, characterized by good structural and functional quality, to which acceptable reduced dimensions of the mouth (microstomia) are added is shown (Figure 35). Not only the modelled images, but also the clinical postoperative clinical images of the previous case (Figure 36 and Figure 37) emphasize these assertions.

At the end of the analysis of the proposed method, we consider it mandatory to adress the way in which it ensures a good final congruence of the lips and the oral cavity. The final congruence is harmoniously achieved through this original method by combining two types of local plasties: The defect of about two-thirds of the lower lip (L.L.) resulting from the oncological ablation of the tumors affecting up to half of the lower lip is initially reduced to one-third of the lower lip by using WY-plasty. The remaining one-third lower lip defect is solved by completing it with the Abbe–Sabattini cross-lip flap whose length could be tailored to one-third of the length of the adjacent upper lip (U.L.).

Finally, by losing the structural tissue of the Abbe–Sabattini cross-lip flap, the final length of the upper lip will remain two-thirds of its original length, being perfectly congruent with the reconstructed lower lip, which completes its remaining one-third length with the other one-third represented by the Abbe flap coming from the upper lip.

Mathematically, in the case of defects of about two-thirds of the lower lip (L.L.) length, the situation is as follows:

(a) L.L. = the remaining one-third length after two-thirds surgical ablation;
(b) Final upper lip (F.U.L.) = the remaining two-thirds length after tailoring the Abbe-Sabattini cross-lip flap which reduces the L.L. by one-third of its length;
(c) Final lower lip (F.L.L.) = the remaining one-third length L.L. + Abbe-Sabattini one-third U.L. = final upper lip (F.U.L.)

In conclusion, the final lower lip (F.L.L.) length will be approximately equal to the final upper lip (F.U.L.) length. In this way, the symmetry of the lips and the oral cavity will be preserved, leading simultaneously to safe esthetic and functional results.

## 3. Results

The proposed method can be used in all situations where a large defect is created in the lower lip (about two-thirds—3.5 fifths of the lip length) and in the conditions of the lack of structural damage to the oral commissures. Although such defects can appear both accidentally (posttraumatic) and following the ablation of some tumoral tissue structures, our work presents the way of applying this innovative method only in the case of patients with large tumors of the lower lip.

This new imagined method for the reconstruction of important defects of the lower lip, consisting of the association of a WY-type plasty (as a method of reducing the primary defect until congruence with an Abbe–Sabattini-type flap) and a classic Abbe–Sabattini cross-lip plasty (as method of canceling a small defect of the lower lip), was applied to several patients.

The criteria for the inclusion of patients in the study were: (a) the existence of a lower lip large tumor; (b) the size of the tumor (maximum one-half–three-fifths of the length of the lower lip); (c) the size of the defect estimated to be created by ablation (about two-thirds–3.5 fifths of the length of the lower lip); (d) a good quality of the structures of the upper lip; (e) a psychological compliance of the patient; (f) lack of metastases in the chin region and in the lower lip.

Based on these selection criteria, we included in the first study, starting from 2021, a group of six patients (four men, two women), aged between 58–76 years. The follow-up period was 12–18 months, during which we also evaluated the long-term result of the application of the method. Only one case presented after 16 months a local recurrence that was removed by minor surgery.

In the following, some of the obtained results will be presented.

Case 1: The patient M.A., 76-year-old female, large lower lip tumor (Figure 36 and Figure 37)

Case 2: The patient B.M., 65-year-old male, large lower lip tumor (Figure 38, Figure 39, Figure 40, Figure 41, Figure 42, Figure 43, Figure 44 and Figure 45).

In order to evaluate the effectiveness of the proposed new method more concretely, it is adequate to compare the aspects of the upper lip, lower lip, and mouth of a patient before and after the surgical procedures are performed, as is presented below (Figure 44 and Figure 45).

## 4. Discussion

Even the proposed innovation appears limited, as these two associated techniques are already well known: we appreciate, however, that the proposed association has a specific applicability in a well-defined situation represented by the reconstruction of large defects of the lower lip (about two-thirds of its length or even more), while maintaining the structural and functional integrity of both oral commissures. Practically, no solution has been described for this type of situation until now. The fact that this proposed innovation simultaneously ensures both the reconstruction of a large defect using only labial structures, as well as the maintenance of the functions ensured by the integrity of the oral commissures, defines it as valuable for this specific type of case. Thus, even if the method associates two well-known techniques utilized for minor reconstruction of the lower lip, it offers the possibility of reconstructing large defects in a way unknown or utilized until now.

We emphasize that the proposed innovative method does not represent a variation of any of the two techniques used, but an association with effects clearly different from those obtained by using them separately.

We consider that a comparative analysis of the advantages or disadvantages of any other similar existing approaches is not correct, especially in the context where none of the known ones ensure the preservation of the structural and functional integrity of both oral commissures.

The new proposed method of lower lip reconstruction is also characterized by the complexity of the implemented strategies, including a multi-level approach. Due to the complexity of this approach, it is mandatory that other specific medical, nursing, and recovery elements must be associated with the surgical interventions.

Even if the plastic surgeon is the determining factor in establishing the lower lip reconstruction strategy, it is mandatory to create a complex team that must include a dermatologist, an oral recovery specialist, a speech therapist, and all those who could provide specific nursing interventions for patients with this type of pathology.

### 4.1. Plastic Surgery Approach

Taking into account the special importance of the functions of the lips, the most important issue in the presence of large defects of the lower lip is the need for a plastic surgeon to choose the most appropriate method for structural and functional reconstruction of lip defects. This requires a general and systematic analysis of the affected structures (skin, vermilion, mucosa, and full thickness), defect size, and involvement of the oral commissure, emphasizing the assessment of full-thickness lip defects. These defects are classified into three categories based on their size: small (<30% of lip length), moderate (30–80%), and extensive (>80%).

The reconstruction of defects limited to the vermilion is usually carried out by advancing the labial mucosa, which is advanced over the orbicularis muscle. Dissection is performed between the mucosa and the underlying musculature. Possible complications include lip thinning due to scar contracture, excessive volume from the over-advancement of the flap, and color mismatch [4]. If simple advancement is not sufficient, other techniques can be used, such as V-Y mucosal advancement flaps, cross-lip mucosal flaps, and tongue flaps, which require a secondary division stage at 14–21 days after the operation [5]. Small lesions can be excised in an elliptical shape and closed primarily, with incisions placed along tension lines for optimal cosmetic results [4].

Concerning perioral cutaneous defects, skin defects are reconstructed with local flaps from the adjacent tissue of the lip, with incisions placed at the borders of the esthetic units [6]. Primary closure is the ideal option, with the long axis of the elliptical excision aligned along the tension lines of the perioral skin, perpendicular to the horizontal axis of the lip [7]. For partial peri-labial defects, either primary suturing or local transposition flaps can be used, keeping the facial musculature intact. Reconstruction of the lower lip may utilize flaps from the chin and submandibular area, with attention to the esthetic distortion of the chin. Large defects of the lower lip can be reconstructed with inferior or bilateral melolabial flaps [4]. Defects of the upper lip can be treated with rotation or transposition flaps, and small defects of the lower portion of the upper lip can be closed with lateral transposition flaps [8].

Full-thickness defects involving less than one-third of the width of the lip can be addressed with primary suturing without compromising function or esthetic appearance. Defects up to 2–2.5 cm can be closed using V, W, or M excisions and V-Y plasties, making sure not to exceed the mentolabial sulcus [2]. After reconstruction, the lip may initially appear tighter, but the skin relaxes over time. In a study, 81% of defects involving less than one-third of the lip were repaired with primary closure, while larger defects were treated with Abbe flaps (6%) and Abbe–Estlander flaps (13%). The majority of patients (81%) had favorable outcomes with minor complications, including hypertrophic scarring (16%) and partial flap necrosis (3%) [9]. A more recently published technique involves creating a myomucosal labial flap, advanced from the side, without affecting the vermillion line. Although this flap requires extensive dissection of the musculature, it provides a better distribution of tension and volume, ultimately resulting in a superior esthetic outcome with minimal complications [10].

Full-thickness defects between 30 and 80% represent the most frequent scenarios and are highly difficult to treat in an appropriate manner.

Thus, large defects of the lips cannot be closed with primary suturing without compromising function and appearance. Various local flaps are used in reconstruction, including the Abbe flap (for central defects), Karapandzic flap (for large defects of the lower lip), and Abbe–Estlander flap (for lateral defects and commissure) [7,11,12]. The Karapandzic flap provides a one-stage reconstruction, but it can lead to a reduction in mouth opening, which can be corrected through commissuroplasty [13]. The Gillis flap is useful for defects near the corner of the mouth but carries the risk of edema-related deformity (the “pincushion” effect) [6]. For large defects of the upper lip, the crescentic peri-alar flap, initially described by Webster, advances the lateral tissue towards the midline, recreating the alar fold and providing an esthetic result without deforming the base of the nasal ala [14,15]. In a study, 33% of cases with defects of up to 80% of the length of the lip were reconstructed with Karapandzic flaps (76%), Bernard–Webster flaps (14%), and peri-alar crescentic flaps (10%). Adverse effects include a reduction in mouth opening (34%), hypertrophic scarring (24%), and flap necrosis (7%) [1].

Total lip defects, referring to the reconstruction of defects that exceed 80% of the length of the lip, are challenging, especially in cases following oncological resections, where the lack of local tissue (mucosa, perioral skin) precludes the use of local flaps. Two main techniques are used: the Bernard–Burrow–Webster flap [16] and free microvascular tissue transfer. The radial forearm free flap (RFF) is the most common method used for total or subtotal lip reconstruction due to its excellent vascularization, pedicle length, and good color match [17]. In cases involving the mandible, the fibular osteocutaneous flap can be used. Specific complications include the loss of oral competence, partial flap necrosis, and total necrosis [18]. A good alternative to the radial forearm flap for the reconstruction of large three-layer lip defects is the anterolateral thigh flap. One of the primary advantages of the anterolateral thigh flap lies specifically in the donor site, because it does not require a split skin graft for coverage and can be closed primarily, unlike the radial forearm flap [18].

Generally, complications of total upper lip reconstructions include hypertrophic scarring, disfigurement, sensory loss, microstomia, and the loss of oral competence. These effects can impact a patient’s ability to eat, making preoperative planning and the development of a therapeutic plan that addresses these issues crucial [18]. Some authors have used the sensate free radial flap, demonstrating recovery of tactile sensation and two-point discrimination at 4 months after the operation [19].

Other reconstruction options described in the literature include the large pectoral muscle flap, deltopectoral flap, cervicodeltopectoral flap, and frontal temporal flap; however, these provide inferior functional and esthetic outcomes [6,20].

Due to the situation created by the impossibility of opening the mouth for 3–4 weeks determined by the Abbe–Estlander cross-lip flap plasty requirements, infections in this area often occur. This is the main factor that imposes a specific antibiotic therapy between the two surgical interventions. A particular concern is identifying multi-antibiotic-resistant pathogens [21], which can sometimes be found in plastic surgery wards.

### 4.2. Dermatological Approach

Most cases of lip tumors are diagnosed by general practitioners during annual or periodic screening. Only some of them benefit from primary specialized diagnoses; in these cases, dermatology specialists are usually involved, either per primam (following the direct presentation of a patient to a specialist consultation) or secondarily via referral by general practitioners [22,23]. Unfortunately, for various reasons, patients with substantial large tumors are not identified as subjects of diagnosis and specialized treatment until late phases, as they are usually sent to be managed by plastic surgeons or oral and maxillofacial surgeons [24].

For this reason, the involvement of dermatologists in the early diagnosis of lip tumors is essential to avoid the appearance of large defects of the labial structures after ablation, which require customized/personalized reconstruction.

### 4.3. Nursing Approach

**Figure 39:**
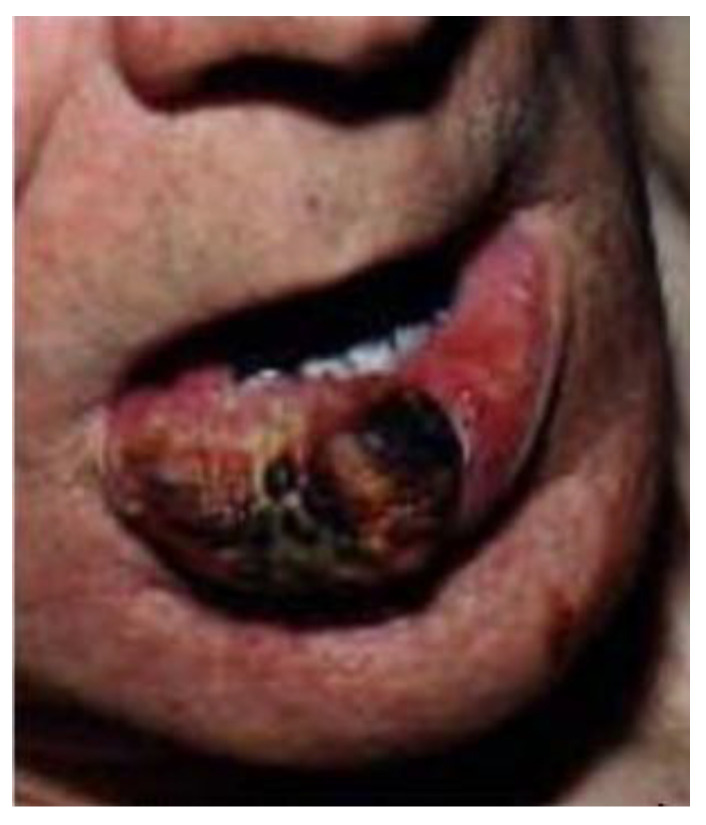
Clinical aspect of the tumor, involving > one-half lower lip length.

**Figure 40:**
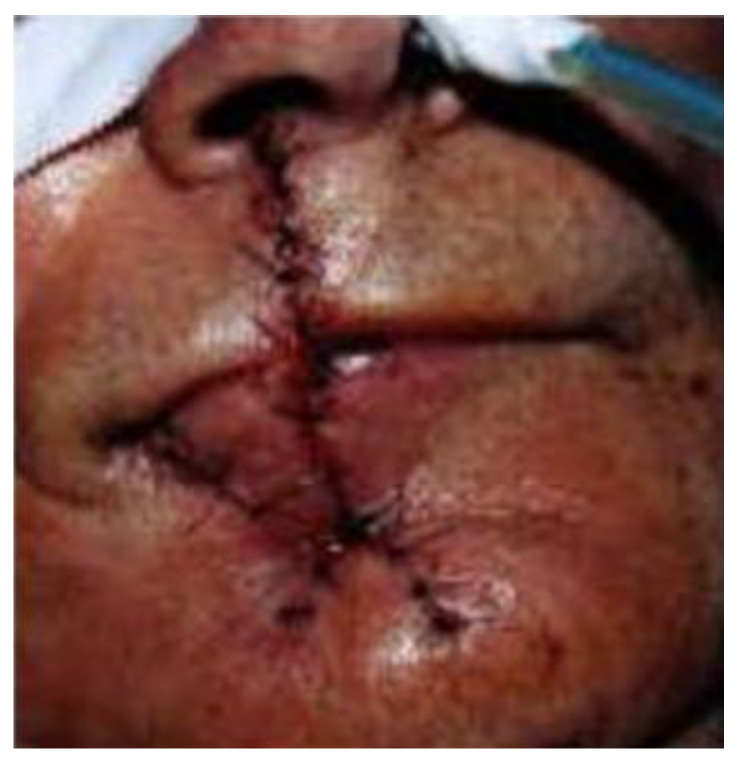
Postoperative aspect (1 day).

**Figure 44:**
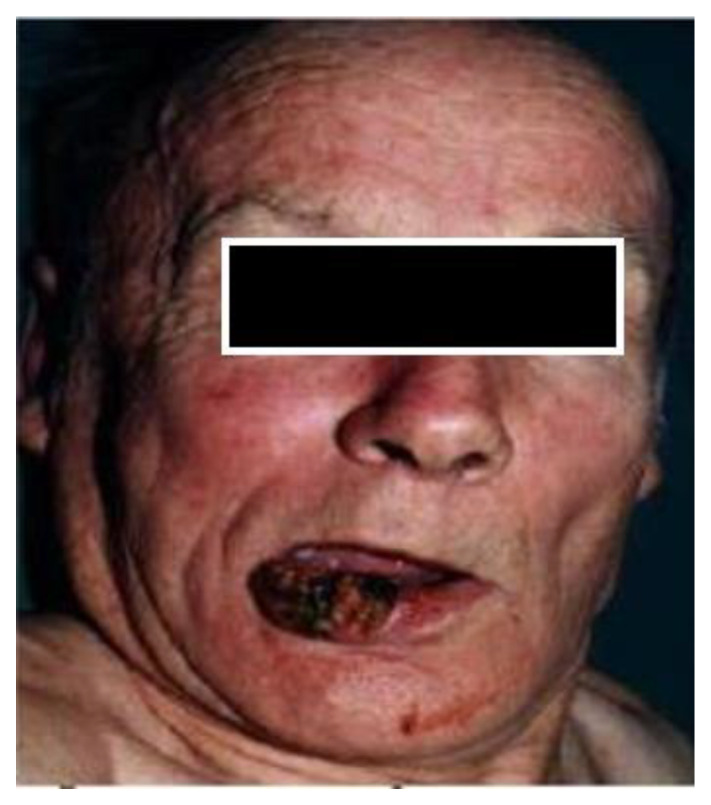
Preoperative situation (B.M., 65-year-old male).

**Figure 45:**
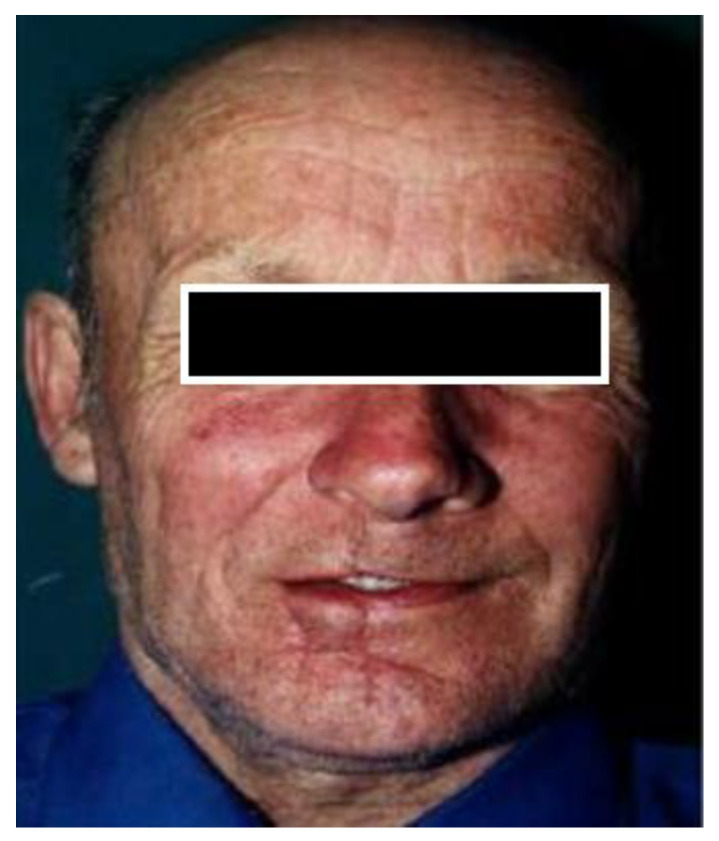
Postoperative situation (B.M., 65-year-old male).

The nursing elements are specifically different for the period following the first stage of the proposed new surgical method compared to those involved after the completion of the second surgical stage. The interval between the two successive interventions is characterized by the most serious nursing problems. Thus, after the first surgical stage, the oral opening is formed by two oral clefts separated by the structure that contains the vascular pedicle of the flap coming from the upper lip, as an element of Abbe–Sabattini cross-lip flap plasty (Figure 30, Figure 31, Figure 39 and Figure 40). In this situation, a patient’s feeding and speech, mastication, and oral hygiene are affected. Wound drains in the neck or near oral wounds will not be necessary, but the care of extra-oral and intra-oral surgical wounds will require a special approach. Eating and drinking will be severely affected, meaning that a patient will not be able to eat or drink normally for almost 3–4 weeks. A nasogastric feeding tube will ensure the feeding process, in which a dietitian will decide how much liquid feed will be needed until a patient can eat and drink normally. The nasogastric feeding tube will be maintained for the entire three/four-week period required between the two surgical stages. Once some primary wound has healed, a patient can gradually increase the amount they drink or eat, so that a dietitian will gradually cut down the liquid feed. Staff must be aware of the special needs of a patient; a call bell will be close by so that a patient can call for help if they need it. A nurse will provide a pen and paper to write down what a patient wants to say. Mobile devices may also help. A nurse may also be able to provide a computer tablet. Some studies demonstrate that the effects of comprehensive (non-conventional) nursing to patients with tumoral oral surgery are remarkable and can promote the recovery of patients and their appearance, decrease postoperative complications, and relieve anxiousness with a high satisfaction rate [25].

### 4.4. Rehabilitation and Speech Therapy Approach

Post-lip tumor surgery physiotherapy and rehabilitation play a crucial role in helping patients regain oral function and facial symmetry and improve their quality of life [26,27]. Rehabilitation typically includes exercises to enhance lip mobility, speech therapy, and strategies to manage any resulting scars or swelling. Physiotherapy must also involve targeted exercises to strengthen the surrounding facial muscles and prevent long-term complications such as reduced lip function or asymmetry. The early initiation of rehabilitation is essential to maximize functional outcomes and promote faster rehabilitation.

### 4.5. Personalized Medicine Approach

The proposed new method cannot be applied in all cases of extensive tumors of the lower lip, allowing only a strictly personalized approach [28,29]. This personalized manner in which to apply this method not only depends on the location or size of a tumor, but, above all, it must account for a patient’s ability to understand the difficulties determined by the almost total loss of function of the oral cavity for a period of 3–4 weeks. The ability of a patient to adapt to major changes in eating and drinking as well as non-verbal communication modalities is essential, and, as a result, must be carefully and systematically analyzed and evaluated before applying the surgical approach.

## 5. Conclusions

This original method is indicated to be mandatorily used in a personalized way only in patients with lower lip tumors in which surgical ablation does not create defects larger than two-thirds of the lower lip and which do not affect the oral commissures, which must mandatorily remain structurally and functionally intact as elements determining a superior result.

This original method has multiple advantages: (a) it does not disorganize the structural and functional elements of the oral commissures, like the majority of other existing methods; (b) it uses only purely labial structures for the reconstruction of the lower lip; (c) it does not require general anesthesia, and can be performed under local anesthesia; (d) technically it is very safe and relatively simple; and (e) it achieves remarkable results, both functionally and esthetically. As a disadvantage, it needs to be performed in two stages, with the second performed after 3–4 weeks, as a condition of Abbe–Sabattini cross-lip plasty.

## Figures and Tables

**Figure 3:**
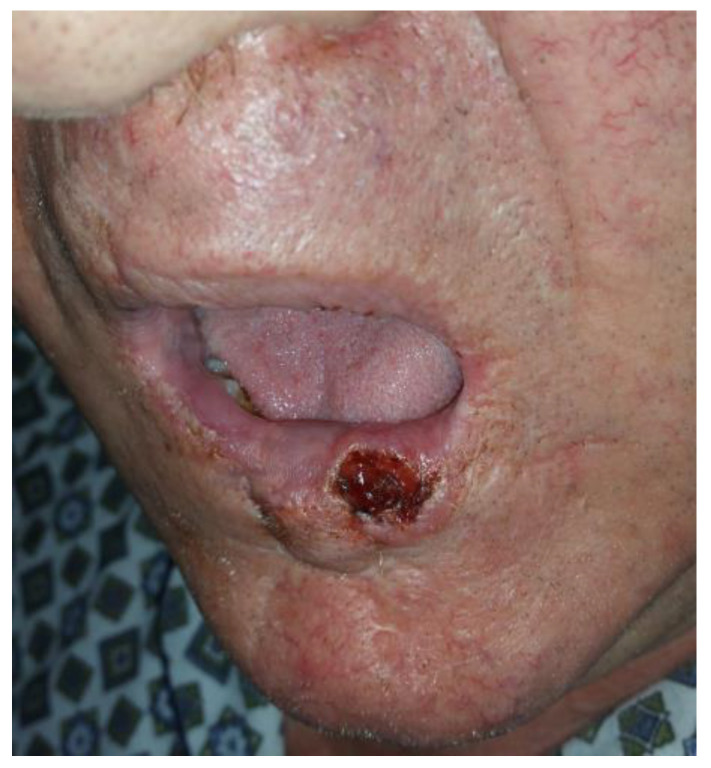
Lower lip malignant tumor requiring the removal > one-third of the lip length (personal case image).

**Figure 4:**
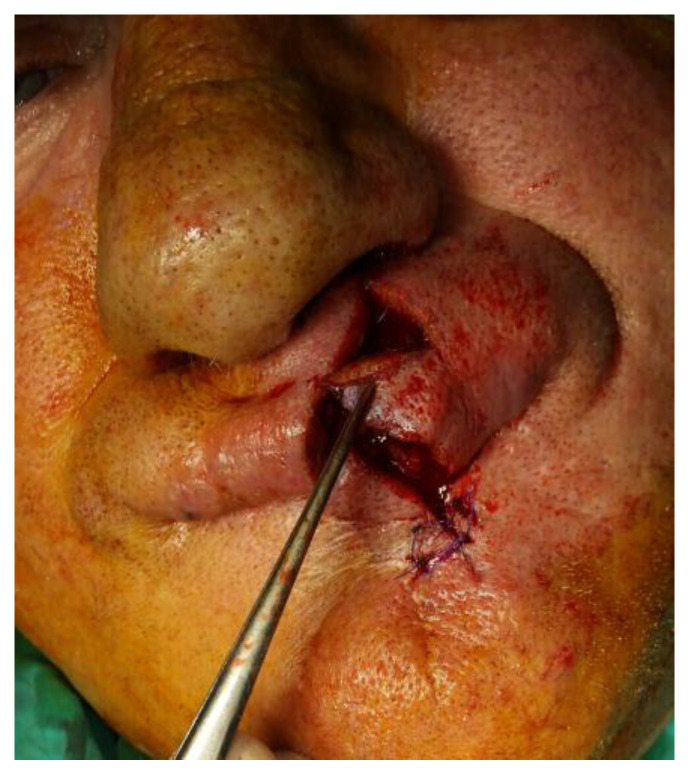
Abbe-Sabattini cross-lip plasty for reconstruction of > one-third of the lip length defect: first step—tailoring the upper lip flap (personal case image).

**Figure 5:**
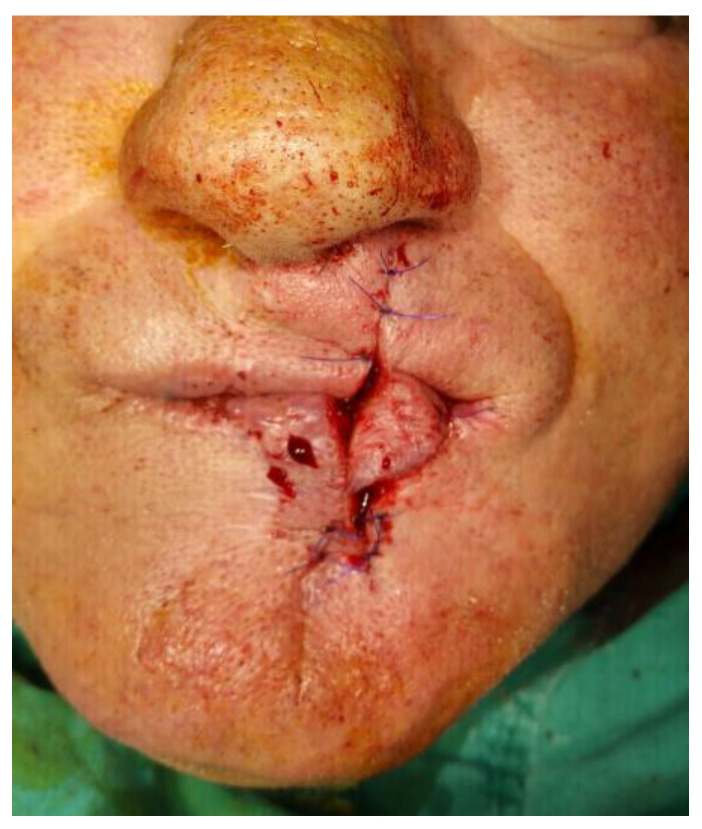
Abbe-Sabattini cross-lip plasty for reconstruction of > one-third of the lip length defect: second step—substituting the lower lip defect (personal case image).

**Figure 6:**
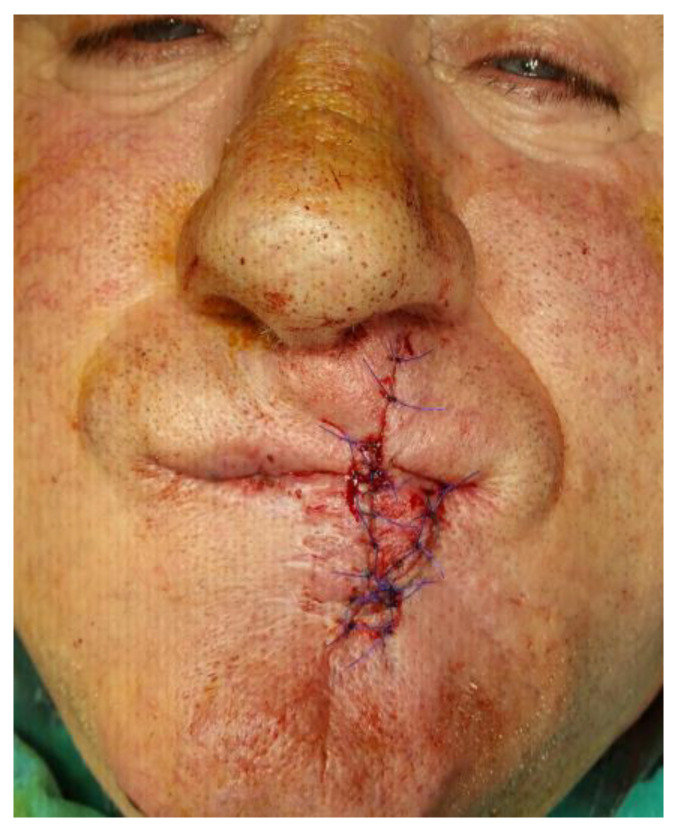
Abbe-Sabattini cross-lip plasty intermediate external result: third step—final aspect (personal case image).

**Figure 7:**
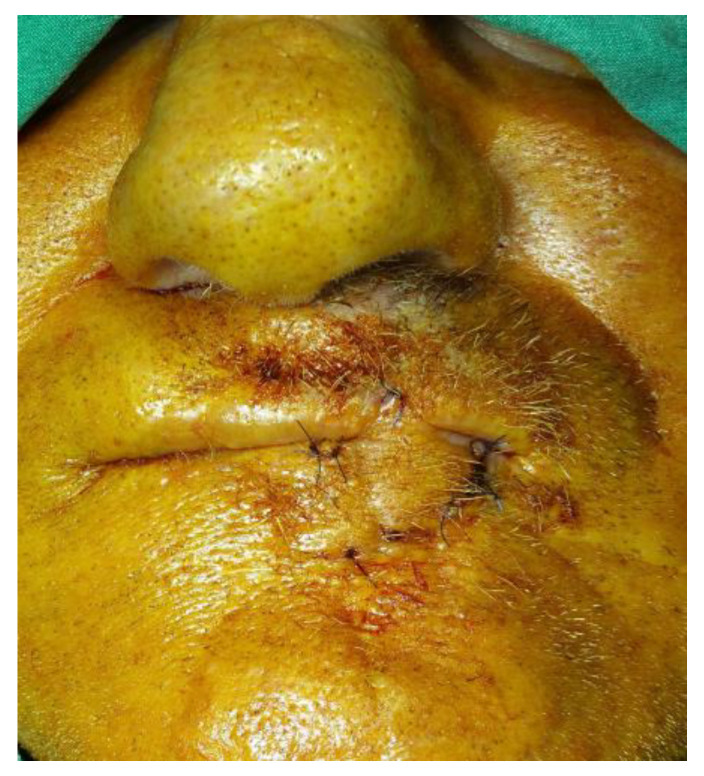
Abbe-Sabattini cross-lip plasty external result after three weeks of local integration of the upper lip flap in the lower lip structure (personal case image).

**Figure 8:**
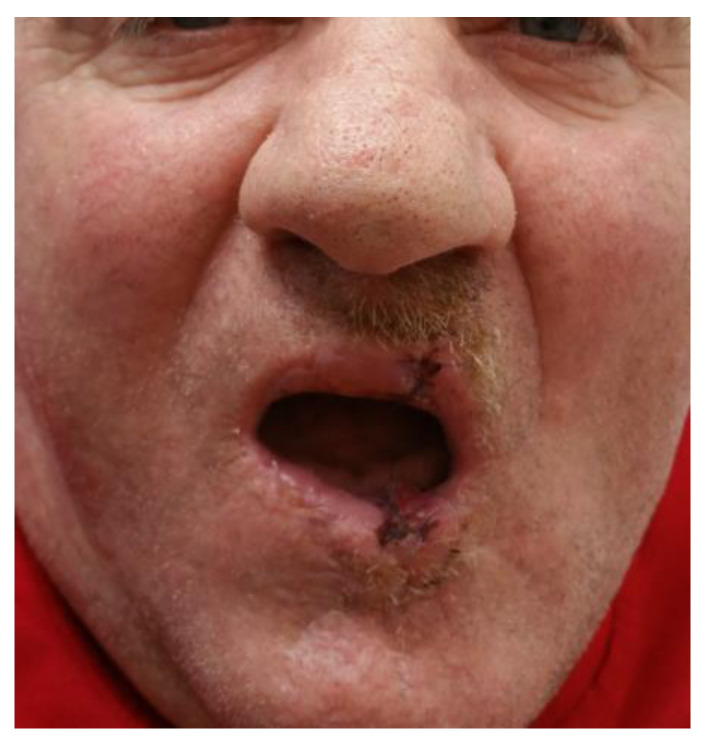
Abbe-Sabattini cross-lip plasty result (final stage) of surgical approach of the 65-year-old male (personal case image).

**Figure 9:**
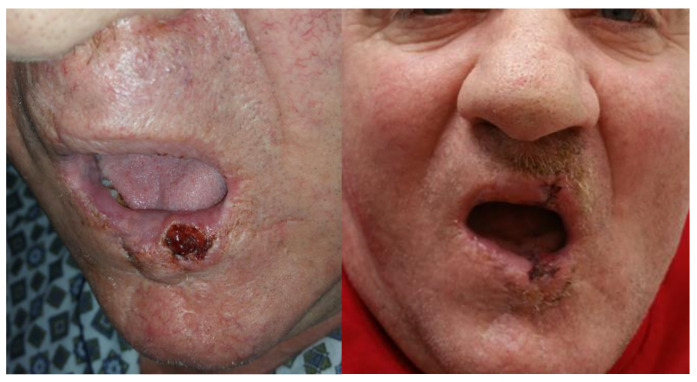
Comparative analysis between the initial vs. final stage of a 65-year-old male patient with lower lip small/medium tumor (personal case image).

**Figure 38:**
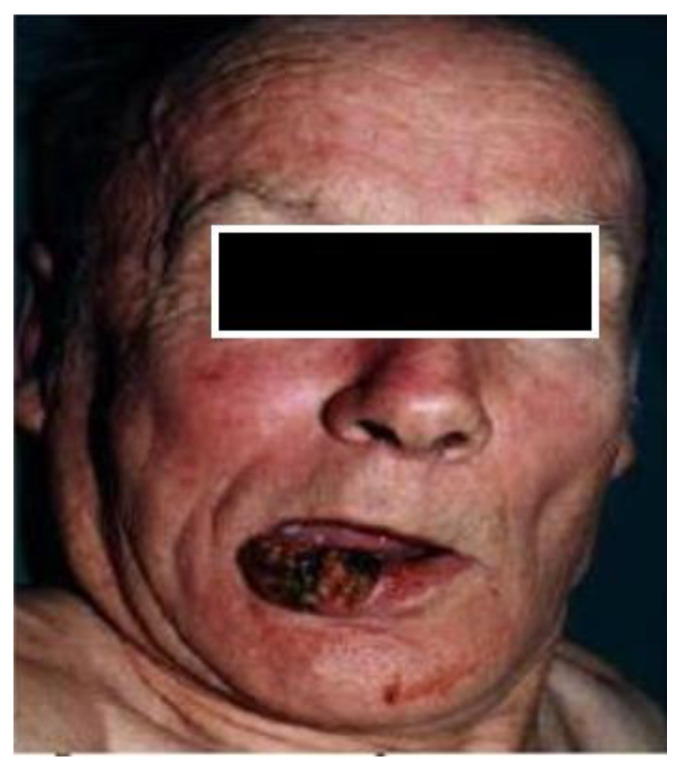
The initial stage of an extensive lower lip tumor (B.M., 65-year-old male).

**Figure 41:**
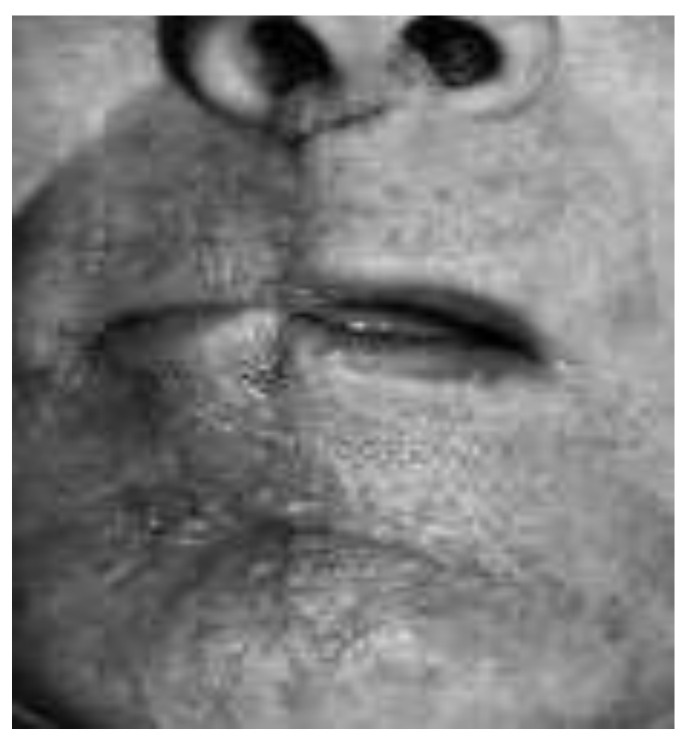
Postoperative aspect (14 days).

**Figure 42:**
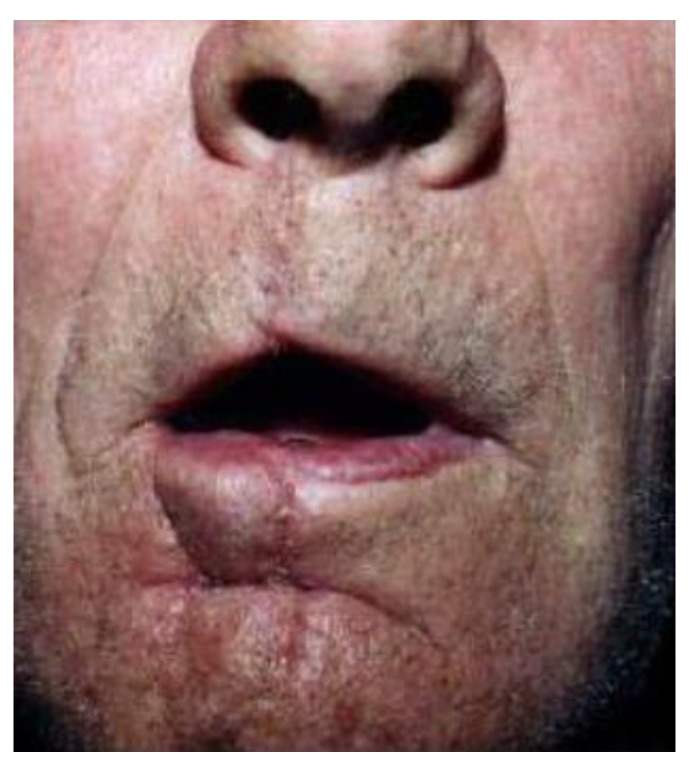
Postoperative final aspect (2 months).

**Figure 43:**
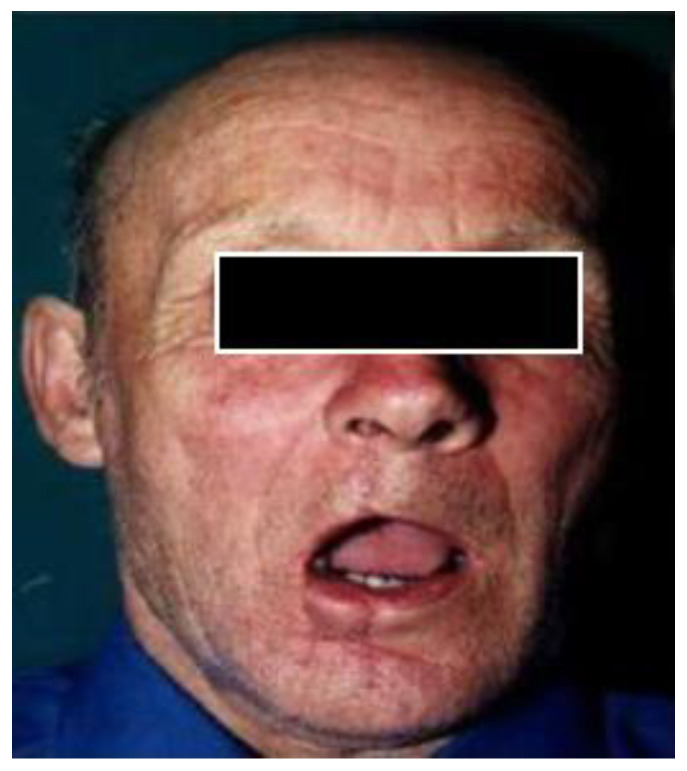
Postoperative final aspect (2 months).

## Data Availability

The data are available if requested.
